# Unique Tubulin-Based Structures in the Zoonotic Apicomplexan Parasite *Cryptosporidium parvum*

**DOI:** 10.3390/microorganisms9091921

**Published:** 2021-09-10

**Authors:** Chenchen Wang, Dongqiang Wang, Jiawen Nie, Xin Gao, Jigang Yin, Guan Zhu

**Affiliations:** Key Laboratory for Zoonoses Research of the Ministry of Education, Institute of Zoonosis, College of Veterinary Medicine, Jilin University, Changchun 130062, China; wcc18@mails.jlu.edu.cn (C.W.); wdq19@mails.jlu.edu.cn (D.W.); niejw18@mails.jlu.edu.cn (J.N.); gaoxin18@mails.jlu.edu.cn (X.G.); yinjg@jlu.edu.cn (J.Y.)

**Keywords:** apicomplexan, *Cryptosporidium*, cytoskeleton, microtubule, immunofluorescence assay

## Abstract

*Cryptosporidium* parasites are known to be highly divergent from other apicomplexan species at evolutionary and biological levels. Here we provide evidence showing that the zoonotic *Cryptosporidium parvum* also differs from other apicomplexans, such as *Toxoplasma gondii*, by possessing only two tubulin-based filamentous structures, rather than an array of subpellicular microtubules. Using an affinity-purified polyclonal antibody against *C. parvum* β-tubulin (CpTubB), we observed a long and a short microtubule that are rigid and stable in the sporozoites and restructured during the intracellular parasite development. In asexual development (merogony), the two restructuring microtubules are present in pairs (one pair per nucleus or merozoites). In sexual developmental stages, tubulin-based structures are detectable only in microgametes, but undetectable in macrogametes. These observations indicate that *C. parvum* parasites use unique microtubule structures that differ from other apicomplexans as part of their cytoskeletal elements.

## 1. Introduction

Microtubules are linear polymers of α- and β-tubulin heterodimers and part of the principal cytoskeletal components in all eukaryotes. Microtubules are involved in a number of cellular functions including maintenance of cell shape, cell motility, intracellular transport and mitosis [1,2]. Apicomplexan parasites are unicellular organisms named after their unique apical complex that is composed of specific cytoskeletal elements and secretory organelles. In a classic structure model of apicomplexan sporozoites, tubulin polymers constitute subpellicular microtubules (aka cortical microtubules) and part of the apical complex, such as conoid and intra-conoid microtubules in some species [3,4,5,6,7]. The number of subpellicular microtubules may vary between species and developmental stages, ranging from 2–3 in the small *Plasmodium falciparum* merozoites to 55–60 in the larger *Plasmodium* ookinetes [4]. In the case of *Toxoplasma gondii* tachyzoites for which cytoskeletons were more thoroughly studied, tubulin-based structures include conoid and a pair of intra-conoid microtubules as part of the apical complex, and 22 evenly distributed subpellicular microtubules in a left-handed spiral that emerge from the apical polar ring (APR) and extend down to two-thirds the length of the tachyzoites [4,5]. Subpellicular and intra-conoid microtubules in *T. gondii* are canonical and composed of 13 tubulin fibers arranged in hollow tubes, whereas the conoid is composed of nine tightly curved and tilted tubulin fibers.

The genus of *Cryptosporidium* is evolutionarily divergent from the coccidia and hematozoa at the base of the Phylum Apicomplexa [8,9,10,11]. However, the cytoskeletal elements in *Cryptosporidium* still remain poorly understood. The majority of the knowledge was derived from earlier morphological observations by electron microscopy (EM), including two “thick central microtubules” in the sporozoites of the intestinal zoonotic species *Cryptosporidium parvum* and up to 10 subpellicular “microtubule-like” structures in the merozoites of the gastric murine species *Cryptosporidium muris* [12,13]. More than 40 longitudinal and spiral “ridges” were also observable under EM. These ridges had a diameter of ~10 nm [13], which was smaller than typical microtubules (~25 nm). They were also located between the plasma membrane and inner membrane complex (IMC; aka alveoli), rather than beneath the IMC for typical subpellicular microtubules (e.g., [13]). Because of their similar arrangement to the subpellicular microtubules in other apicomplexans, the ridges were sometimes labeled as microtubules [14], but the molecular nature of the “ridges” remains to be defined.

The molecular nature of “microtubules” or tubulin-based structures were less defined in *C. parvum*. There has been only one study of indirect immunofluorescence assay (IFA) and immunogold electron microscopy (IEM) using commercially available monoclonal antibodies against sea urchin α-tubulin and rat β-tubulin. The anti-α-tubulin antibody recognized a 50-kDa protein band in Western blot analysis and labeled the apex of the sporozoite (IEM) plus one or two filamantous structures of sporozoites (IFA and IEM). The anti-β-tubulin antibody failed in Western blot analysis, but nonetheless produced strong signals at the tip of the sporozoites. Although not definitive, the authors concluded that tubulin polymers formed “the apical rings, the electron-dense collar in the apical region and two central microtubules” [13].

Considering that cytoskeletons are essential to all eukaryotic cells and tubulins have been found to be an excellent drug target for developing anti-cryptosporidial therapeutics [15], we decided to clarify the tubulin-based structures in the zoonotic species *C. parvum*. More specifically, a rabbit polyclonal antibody against a short peptide specific to the *C. parvum* β-tubulin (CpTubB) was developed, affinity-purified and validated by Western blot analysis. Using this antibody, we were able to label tubulin-based structures in various developmental stages throughout the parasite lifecycle. We observed a long and a short microtubule in the parasite sporozoites that were rigid and underwent dynamic morphological changes during the invasion and intracellular development.

## 2. Materials and Methods

### 2.1. Antibody Development and Affinity-Purification

Anti-CpTubB antibody: Tubulin-based structures are composed of α- and β-tubulin heterodimers that can be labeled by an antibody specific to either α- or β-tubulin. In this study, an anti-CpTubB polyclonal antibody was developed against a short synthetic peptide (^433^DEYPDDEHHI^442^) unique to β-tubulins in *C. parvum* and *Cryptosporidium hominis*. Peptide was synthesized by ChinaPeptides Company (Shanghai, China), linked to keyhole limpet hemocyanin (KLH) via maleimidobenzoyl-N-hydroxysuccinimide ester in-house [16], and used to immunize two specific pathogen-free rabbits using a standard antibody production protocol [17]. Briefly, rabbits were administrated by subcutaneous injections of KLH-linked peptide emulsified with Freund’s complete (first injection; 300 μg) and incomplete adjuvants (subsequent three injections; 150 μg each) in a two-week interval. Pre-immune sera and antisera were collected prior to the first injection and two weeks after the last one. The animal use protocol was reviewed and approved by the Institute Committee for Biosafety and Ethics for Animal Use, Jilin University (AUP number IZ-2019-084; approval on 10/10/2019).

The antibody titers were determined by ELISA and coated with synthetic peptide conjugated with BSA (0.25 μg/well). Specific antibody was affinity-purified by a nitrocellulose membrane-based protocol with slight modification [18]. Briefly, 100 μg peptide dissolved in 300 μL ddH_2_O was immobilized to the membrane (~1.0 cm^2^), followed by blocking with 5% skim milk-TBST buffer and washed three times in TBST, incubation with 4 mL of 1:20 diluted antisera for1 h at room temperature and overnight at 4 °C, five washes with TBST and elution with 1 mL glycine elution buffer (GBST; 0.2 M glycine, 0.15 M NaCl, 0.05% Tween-20, pH 2.7), and immediate neutralization of eluted antibody with 50 μL of 1.0 M Tris buffer as described [18]. After overnight dialysis against PBS, affinity-purified antibody was used immediately or stored at −20 °C until use. Secondary antibody used was horseradish peroxidase (HRP)-conjugated goat anti-rabbit IgG(H+L) antibody (Immunoway, Plano, TX, USA) for Western blot analysis, or Alexa Fluor 488 goat anti-rabbit IgG (Invitrogen, Waltham, MA, USA) for IFA.

### 2.2. Preparation of Parasite Materials

Parasite: A strain of *C. parvum* (subtype IIaA17G2R1 at the *gp60* locus) was propagated in-house in calves. Oocysts were purified from calf feces using a standard sucrose/CsCl gradient centrifugation protocol [19], and stored in PBS containing penicillin (10^4^ unit/mL) and streptomycin (10^4^ μg/mL) at 4 °C until use. 

Extracellular stages (oocysts and sporozoites): Prior to experiments, oocysts were surface-sterilized by suspension in 4% (*v*/*v*) sodium hypochlorite on ice for 5 min, followed by five or more washes in PBS. The viability of oocysts was tested by an in vitro excystation assay as described [20], and only those with >80% excystation rates were used in infection experiments. In vitro excystation protocol was also used to prepare free sporozoites. In some experiments, ruptured sporozoites were prepared by a hypotonic treatment of sporozoites in 0.2× PBS with vigorous vortex for 1 min, followed by one cycle of freeze and thaw in liquid nitrogen and 37 °C waterbath. Specimens were lysed in appropriate lysis buffers for isolating RNA or proteins, or fixed for IFA as specified below.

Intracellular stages: The in vitro cultivation of *C. parvum* was hosted in HCT-8 cells (a human ileocecal colorectal adenocarcinoma cell line; ATCC # CCL-244) as described [21,22]. HCT-8 cells were routinely cultured in RPMI-1640 medium containing 10% fetal bovine serum (FBS) and penicillin (10^4^ unit/mL)/streptomycin (10^4^ μg/mL) at 37 °C under 5% CO_2_ atmosphere. Prior to infection, HCT-8 cells were seeded into 48-well plates and allowed to grow overnight until cell monolayers reached ~90% confluence. For IFA experiments, round glass coverslips coated with poly-L-lysin were placed into plates to support the cell growth.

Invading sporozoites were prepared by adding excysted sporozoites into the plates containing HCT-8 cell monolayers (~2 × 10^6^ sporozoites per well) twice in a 15-min interval to produce specimens containing sporozoites invading host cells for ~45 to 60 min. Other intracellular stages of *C. parvum* were prepared by infecting HCT-8 cell monolayers for specified time points. Briefly, clean oocysts were added into the plates and allowed for excystation and invasion for 2 h. After three washes with FBS-free culture medium, the invaded parasites were allowed to develop for specified times for preparation of cell lysates for qRT-PCR or fixation for IFA.

### 2.3. qRT-PCR Analysis of CpTubA and CpTubB Gene Transcripts

To better understand the molecular features of CpTubA and CpTubB, we also analyzed their gene expression levels in various parasite developmental stages. The relative levels of transcripts of *CpTubA* gene (CryptoDB gene ID: cgd4_2860; for clarity, gene names are written in italics) and *CpTubB* gene (cgd6_4760) during the parasite life cycle were detected by qRT-PCR using previously reported primer pairs: 5′-ACA GAG GTG ATG TTG TTC CAA A-3′ and 5′-TTA ATT CCA CAT TTG AAG CCT G-3′ for *CpTubA* and 5′-AGC CCT ACA ATG CAA CCT TAT C-3′ and 5′-ACA AGT TAC GCC AGA CAT AGC A-3′ for *CpTubB* [23]. The levels of *C. parvum* 18S rRNA were also detected for normalization using primers 5′-TAG AGA TTG GAG GTT GTT CCT-3′ and 5′-CTC CAC CAA CTA AGA ACG GCC-3′ [23,24]. For comparison and quality control, the transcript of previously reported *CpLDH* gene was also detected in parallel [25,26].

Cell lysates were prepared from oocysts, sporozoites and intracellular parasites at specified post-infection time points to obtain total RNA using an iScript qRT-PCR sample preparation reagent (lysis buffer) (Bio-Rad Laboratories, Hercules, CA), which were diluted as described for use as templates for qRT-PCR; qRT-PCR was carried out using HiScript II One-Step qRT-PCR SYBR^®^ Green Kit (Vazyme Biotech, Nanjing, China) as described [20,22]. Each reaction in 20 μL final volume contained 0.2 μM of each primer, 1.0 μL One Step SYBR enzyme mix, 10 μL SYBR Green mix, and 0.4 μL ROX reference dye 1 (50×), 0.2 ng of total RNA isolated from oocysts/sporozoites or 15 ng total RNA isolated from intracellular parasites. Reactions were performed in a StepOnePlus thermal cycler (ThermoFisher, Waltham, PA). Thermal cycling started with 50 °C for 3 min to synthesize cDNA, followed by incubation at 95 °C for 30 s for inactivate the reverse transcriptase and 40 cycles at 95 °C for 10 s and 60 °C for 30 s to produce amplicons. At least two technical replicated qRT-PCR reactions were performed for each sample. The relative levels of transcripts were calculated using a 2^−∆∆CT^ formula as described [24].

### 2.4. Western Blot Analysis

Freshly excysted sporozoites were added into 1× SDS-PAGE sample buffer, heated for 5 min at 95 °C, and fractionated by 10% SDS-PAGE (4 × 10^7^ sporozoites per lane), followed by the transfer of proteins onto nitrocellulose membranes. The blots were incubated for 1 h in a blocking buffer containing 5% skim milk in TBST (10 mM Tris-HCl (pH 7.5), 150 mM NaCl and 0.05% Tween-20), washed three times in TBST, incubated for 1 h with primary antibodies prepared in 5% skim milk in TBST (1:50 dilution for affinity-purified rabbit anti-CpTubB antibody), washed five times with TBST, and incubated again for 1 h with HRP-conjugated goat anti-rabbit IgG antibody (Immunoway; 1:10,000 dilution). After five final washes with TBST, the blots were developed using an enhanced chemiluminescence reagent and visualized in an UVP ChemStudio (Analytik Jena, Upland, CA, USA). All procedures were conducted at room temperature unless specified, and all washes were performed for 5 min.

### 2.5. Indirect Immunofluorescence Assay (IFA)

Various developmental stages of *C. parvum* were prepared as described above and fixed for 30 min in 4% paraformaldehyde prepared in PBS. Oocysts and excysted sporozoites were fixed in suspension, while host cells containing intracellular parasites were fixed as monolayers on coverslips. Oocysts after fixation were subjected to additional three freeze/thaw cycles in liquid nitrogen and 37 °C water bath (2 min each). All samples fixed in suspension were centrifuged to remove fixative, washed three times in PBS, resuspended in PBS at appropriate concentrations, and applied onto glass slides treated with 0.1 mg/mL poly-L-lysin. Samples on slides were subjected to semi-dry in air for 30 min. All samples were permeabilized in 0.1% Triton X-100 for 5 min, followed by three washes in PBS, blocking in 3% BSA for 1 h, and three washes in PBS.

To test the sensitivity of sporozoite microtubules to inhibitors, colchicine (50 μM; microtubule assembly inhibitor) and paclitaxel (50 μM; disassembly inhibitors) were included during the excystation process (vs. 1% dimethyl sulfoxide (DMSO) diluent control). After excystation for 45 min, samples were subjected to the same wash, fixation and permeabilization procedures as described above.

Specimens were then incubated with primary antibodies prepared in PBS (1:3 dilution for affinity-purified rabbit anti-CpTubB antibody), followed by five washes and incubation with Alexa Fluor 488-conjugated goat anti-rabbit IgG (1:2000 dilution). After final three washes, specimens were stained with DAPI (1 μg/mL in PBS) for 5 min, washed five times with PBS, mounted with an antifade mounting medium (Beyotime, Shanghai, China), and examined under a BX53 research fluorescence microscope (Olympus Corp., Tokyo, Japan) equipped with appropriate filter sets. Microscopic images in TIFF format were captured with an Olympus DP72 camera and stored in TIFF format. Linear adjustment of levels was performed with Adobe Photoshop (v22.4; Adobe Inc., San Jose, CA, USA) to better visualize the structural features. Images were annotated with Adobe Illustrator (v25.3; Adobe Inc., San Jose, CA, USA).

### 2.6. Phylogenetic Analysis of Apicomplexan α-, β- and γ-tubulin Proteins

*Cryptosporidium* α-, β- and γ-tubulin protein sequences were used as queries to search the NCBI reference protein sequence database (https://blast.ncbi.nlm.nih.gov; last access on 8 September 2021) for alveolate orthologs by the BLASTP algorithm with E-value cutoff set at 10E-40. Protein sequences were retrieved and subjected to multiple rounds of multiple sequence alignment using MUSCLE (v3.8.31) (http://www.drive5.com/muscle/; last access on 8 September 2021). Based on the alignments and rough neighbor joining trees, incomplete and redundant sequences were removed to give a dataset containing 90 representative sequences, i.e., 38, 28 and 24 sequences for α-, β- and γ-tubulin, respectively. After removing positions with gaps and alignment ambiguousness, a final of 395 amino acid positions in the final dataset were used for subsequent phylogenetic analysis.

Phylogenetic trees were reconstructed by a Bayesian inference method using MrBayes (v3.2.6) (http://nbisweden.github.io/MrBayes/; last access on 8 September 2021). Amino acid substitutions used a mixed model to allow sampling across all amino acid rate matrices available in the program, with the consideration of the proportion of invariable sites and four discrete rate categories of gamma distribution across sites. A total of 10^6^ generation of tree searches were performed with two independent searches running with four chains. Trees were sampled in every 1000 generations of run. Consensus trees were summarized with posterior probabilities from the bottom 75% of the sampled trees, displayed with FigTree (v1.4.4) (http://tree.bio.ed.ac.uk/software/figtree/; last access on 8 September 2021) and annotated with Adobe Illustrator (v25.3). 

## 3. Results and Discussion

### 3.1. The α- and β-tubulin Genes in C. Parvum Are Differentially Expressed during the Parasite Life Cycle

**Figure 1:**
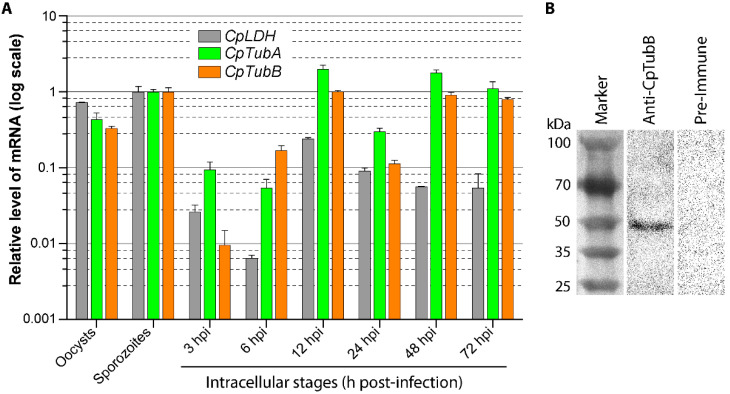
Detection of *Cryptosporidium parvum* α- and β-tubulin (*CpTubA* and *CpTubB*) gene transcripts and CpTubB protein. (**A**) Relative level of *CpTubA* and *CpTubB* transcripts in various parasite developmental stages obtained from in vitro culture as determined by qRT-PCR. A previously reported parasite lactate dehydrogenase gene (*CpLDH*) was known for its expression pattern [25,26], which was assayed in parallel for comparison and as a quality control. The levels *C. parvum* 18S rRNA were used for normalization, and those at the sporozoite stage were used as the baseline. (**B**) Western blot detection of CpTubB protein from the sporozoite crude extracts using an affinity-purified anti-CpTubB polyclonal antibody raised in rabbits. The pre-immune serum was subjected to the same affinity purification procedure and used as control.

The transcripts of α- and β-tubulin genes in *C. parvum* (*CpTubA* and *CpTubB*) were detectable in oocysts, sporozoites and intracellular developmental stages from 3 to 72 h post-infection (hpi) time points (Figure 1A). The expression of *CpTubA* and *CpTubB* genes followed a similar pattern, in which relatively consistent high levels of transcripts were detected in oocysts, sporozoites and intracellular stages between 12 to 72 hpi, while the lowest levels were detected in the intracellular stage at 3 and 6 hpi. The intracellular development of *C. parvum* were more synchronized from 3 to 12 hpi which would represent development from newly formed trophozoites to the developing type I meronts (vs. coexistence of mature meronts and released and invading merozoites at 24 hpi or later), and which made the data derived from 3 to 12 hpi more comparable. Therefore, up to 1 to 2 orders of magnitude of lower levels of *CpTubA* and *CpTubB* transcripts at 3 to 6 hpi (vs. 12 hpi) (Figure 1A) were indicative that the expression of the two parasite tubulin genes was significantly suppressed in the very early stages of intracellular development (i.e., one-nucleus trophozoites).

### 3.2. Tubulin Polymers Form Two Microtubular Filaments in the C. parvum Sporozoites Differing from Those in Other Apicomplexans

**Figure 2:**
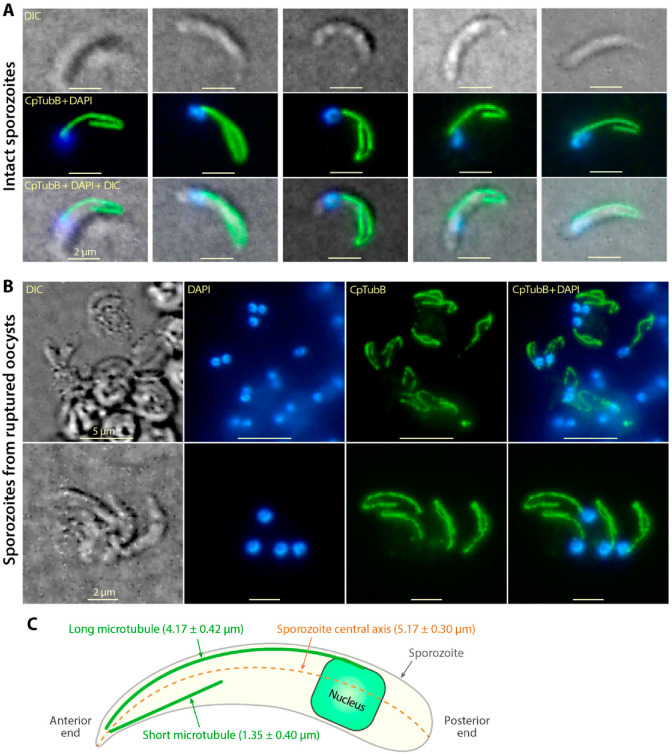
Indirect immunofluorescence assay (IFA) detection of tubulin-based structures in the sporozoites of *Cryptosporidium parvum* using affinity-purified anti-CpTubB antibody (green) and counterstained with 4′,6-diamidino-2-phenylindole (DAPI) (blue). (**A**) IFA of CpTubB in excysted intact sporozoites. (**B**) IFA of CpTubB in unexcysted sporozoites, in which anti-CpTubB antibody was only able to label sporozoites retrieved from oocysts after a freeze-and-thaw treatment. (**C**) Lengths of the excysted sporozoites (orange), long and short microtubules (green) (*n* = 30). DIC, differential interference contrast microscopy.

In order to investigate tubulin-based cytoskeletal structures, a polyclonal antibody was raised in rabbits and affinity-purified. Western blot analysis confirmed the specificity of the affinity-purified anti-CpTubB antibody as it recognized a single protein band from crude extracts in sporozoites (Figure 1B). Surprisingly, IFA using this antibody labeled two microtubule filaments in the sporozoites of *C. parvum* (Figure 2A), rather than multiple subpellicular microtubules as seen in other apicomplexans [3,4,7,27]. Both microtubules emerged from the apical region, and one of them was much longer than the other one (Figure 2A). The long microtubule appeared to be subpellicular, as it was typically shaped in a segmental arch and distributed along with the convex side of the banana-shaped sporozoites, like a “backbone” for a sporozoite, whereas the short microtubule was usually straight, suggesting that it was not subpellicular (Figure 2A).

The sporozoites in oocysts possessed the same two microtubule filaments, which could only be clearly observed after the sporozoites were retrieved from ruptured oocysts by a freeze-and-thaw treatment (Figure 2B). The length of the long microtubule in excysted sporozoites was relatively consistent (mean length = 4.17 ± 0.42 μm; vs. 5.17 ± 0.30 μm for the length of sporozoites) and extended to the posterior region (80.7% of the sporozoite length) where the nuclei were located. The lengths of the short microtubule were more variable (mean length = 1.35 ± 0.40 μm), ranging from ~20% to 50% of the long one (mean ratio = 32.4% ± 9.7%) (Figure 2C).

**Figure 3:**
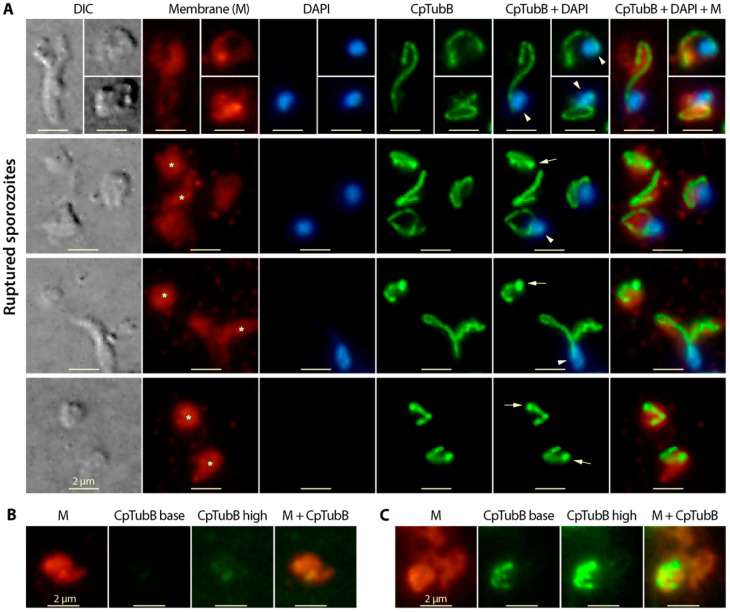
IFA detection of tubulin-based structures in the ruptured sporozoites of *Cryptosporidium parvum* by anti-CpTubB antibody (green) and 4′,6-diamidino-2-phenylindole (DAPI) for nuclei (blue). (**A**) IFA of CpTubB ruptured sporozoites and membrane pieces produced by a hypotonic vortex treatment. Membranes (M) were labeled using a monoclonal antibody against a *C. parvum* membrane protein (red). Asterisks indicate membrane pieces lacking nuclei stained with DAPI. Arrowheads indicate ball-shaped ends of shrunk microtubules. Arrows indicate nuclei located at the posterior end of the long microtubule. (**B**,**C**) IFA of CpTubB in membrane pieces (M) lacking associated nuclei in the absence (**B**) or presence (**C**) of the two microtubules. The levels of CpTubB-derived fluorescence signals were shown as baseline signals (marked by “CpTubB base”) or adjusted to higher (marked by “CpTubB high”) for visualizing potential other types of membrane associated tubulin-based filaments with weak signals.

As mentioned in the introduction, multiple subpellicular microtubules were described or implied by the presence of >40 longitudinal and spiral ridges in earlier ultrastructural studies [13,14]. However, these structures were not recognized by the anti-CpTubB antibody in this study. It was possible that the strong fluorescent signals from the two microtubules observed here masked the relatively weak signals from other tubulin-based structures if present. To rule out this possibility, we prepared ruptured membrane pieces from sporozoites by suspended excysted sporozoites in a hypotonic solution (0.2× PBS), followed by vigorous vortex and a freeze/thaw cycle. This hypotonic treatment allowed better separation of the two microtubules from other possible subpellicular microtubules (Figure 3). In this experiment, the affinity-purified anti-CpTubB antibody again labeled the long and short microtubes only, but not any other filamentous structures (Figure 3A). On the “blank” membrane pieces lacking associated nuclei, no fluorescent filamentous structures were observable after the images were artificially overexposed by adjusting the signal levels in Photoshop (Figure 3B,C).

These observations concluded the presence of a long and a short microtubule in the sporozoites of *C. parvum* and the lack of other type of tubulin-based structures that could be recognizable by the anti-CpTubB antibody used in this study.

### 3.3. The Two C. Parvum Microtubules in the Sporozoite Stage Are Rigid, and the Long Microtubule Appeared to Be Associated with the Nucleus

The two microtubules were well preserved even after sporozoites were ruptured, although they might be shortened and/or winding back towards the anterior end (Figure 3). Some microtubular filaments, particularly the long one, might be shrunk into a ball-like appearance at the posterior ends (indicated by arrows in Figure 3A). The rupture of sporozoites by hypotonic vortex was vigorous, resulting in the formation of membrane pieces in varied shapes, including some pieces without nuclei (marked by asterisks in Figure 3A). However, whenever nuclei were present, they were usually located at the posterior ends of the long microtubule (marked by arrowheads in Figure 3A), which implied an association of the nuclei with the long microtubule. The association was reasonably strong as it was resistant to certain levels of physical force produced by hypotonic vortex.

The stability of the two microtubules in the sporozoites was also confirmed by treating the parasite oocysts during excystation with colchicine and paclitaxel (microtubule assembly and disassembly inhibitors), in which both microtubules in sporozoites after excystation showed no or little changes in appearances and lengths between control and treated samples (Table 1). Microtubules are known to be more rigid and stable in apicomplexans such as *T. gondii* than those in other eukaryotes, probably due to their post-translational modifications and/or affixation with microtubule associated proteins [5,28]. Our observations confirmed that *Cryptosporidium* microtubules were also rigid and stable as seen in other apicomplexans.

### 3.4. The Two Microtubules in C. Parvum Are Restructured during the Invasion of Sporozoites and Its Transformation into Trophozoites

**Figure 4:**
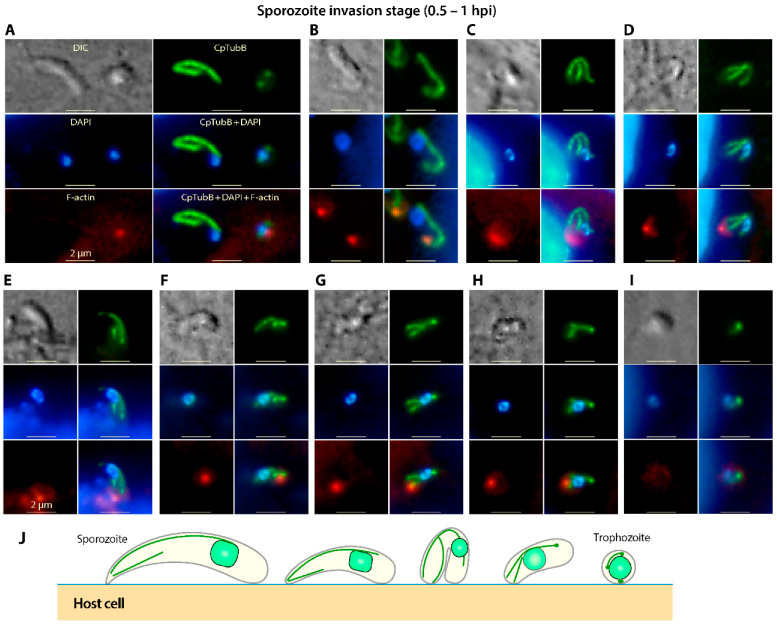
IFA detection of tubulin-based structures in *Cryptosporidium parvum* during the invasion of the host cell by sporozoites and its development into trophozoites by anti-CpTubB antibody (green). Nuclei were counterstained with 4′,6-diamidino-2-phenylindole (DAPI) (blue). Panels (**A**–**I**) represent various stages of transformation of banana-shaped sporozoites into small round trophozoites. Aggregated host cell F-actin at the infection site was stained with phalloidin-rhodamine (red). Panel (**J**) illustrates the morphological changes of the two microtubules in a sporozoite during invasion. For simplicty, the formation of parasitophorous vacuole membrane during the sporozoite invasion was not illustrated here. DIC, differential interference contrast microscopy.

The two microtubules remain clearly visible in sporozoites during the invasion (Figure 4). Upon the attachment to host cells, some sporozoites might be bent from the middle as indicated by the shape of the long microtubules (Figure 4C,D), suggesting plasticity of the sporozoites and the microtubule. The bending of sporozoites for a certain time after their attachment to host cells was also reported by other investigators very recently [29], suggesting that this was not an artifact or incidental behavior. During the invasion process, both the long and short microtubules became shortened while the parasites underwent transformation from banana-shaped sporozoites to round trophozoites. Eventually, the two microtubules appeared as two or three bright dots in the tiny trophozoites (Figure 4A–I).

It was noticeable that the nucleus in an invading sporozoite gradually moved from the posterior end towards the anterior end, making it appear to be moving along the long microtubule (Figure 4). Microtubules are known to be involved in intracellular trafficking, including the trafficking of organelles such as nuclei [30,31,32,33]. However, whether the long microtubule is truly involved in anchoring and trafficking of nuclei in the sporozoites can only be validated by further investigation, including the identification and confirmation of linker molecules between the microtubule and nuclear envelope in the parasite.

The short microtubule in an invading sporozoite was rearranged and moved towards the infection site, making it appear near the aggregated host cell F-actin that were labeled with rhodamine-phalloidin as red dots in Figure 4A–I. This observation leads us to speculate that the short microtubule might possibly be involved in the anchoring of the sporozoites at the infection sites during invasion of the host cell.

### 3.5. The Two Microtubules of C. parvum Are Present in the Asexual Developmental Stages (Merogony) and Rearranged during Parasite Development

**Figure 5:**
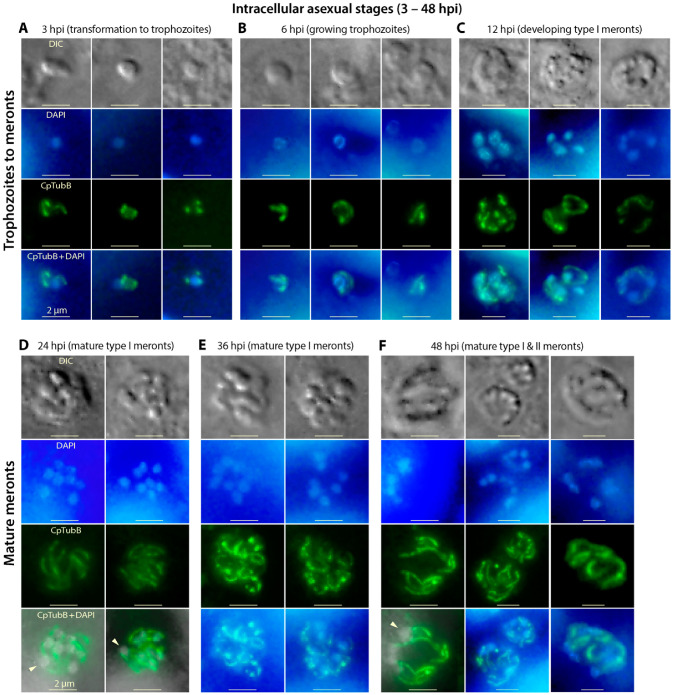
IFA detection of tubulin-based structures in *Cryptosporidium parvum* during various intracellular asexual developmental stages. Panels (**A**–**F**) show the intracellular parasites at 3 to 48 h post-infection (hpi), representing transforming sporozoites and transformed trophozoites with one nucleus (**A**,**B**), developing type I meronts containing four nuclei (**C**) and mature type I and II meronts containing eight or four nuclei, respectively (**D**–**F**). Microtubules were labeled by anti-CpTubB antibody (green), while nuclei were stained by 4′,6-diamidino-2-phenylindole (DAPI) (blue). In the merged images of panels (**D**,**F**), blue nuclei were converted to grey for better visualization (pointed by arrowheads). DIC, differential interference contrast microscopy.

The shortened long and short microtubules are indistinguishable in the trophozoite stage (Figure 5A). They grew longer during the development of trophozoites that contained a single growing nucleus (Figure 5B). In the multi-nuclear meronts, the two microtubules became more apparent, but their structures could not be well resolved due to the limitation of microscopic resolution (Figure 5C–F). However, the parasite microtubules were apparently present in pairs of long and short filaments (one pair per nucleus or per merozoite). In mature meronts containing developed merozoites, nuclei typically appeared at the posterior end of the long microtubules as seen in the sporozoites (Figure 5D,F). *Toxoplasma* microtubules are documented to play a critical role the separation of nuclei and enclosed chromosomes during the closed mitosis [34]. Whether and how microtubules are formed and/or reorganized to participate in the schizogonic cell division in *Cryptosporidium* parasites remains an intriguing question for further investigation.

### 3.6. Tubulin-Based Structures Were Present in Microgametes, but Appear Absent in Macrogametes in the Sexual Development of C. parvum

**Figure 6:**
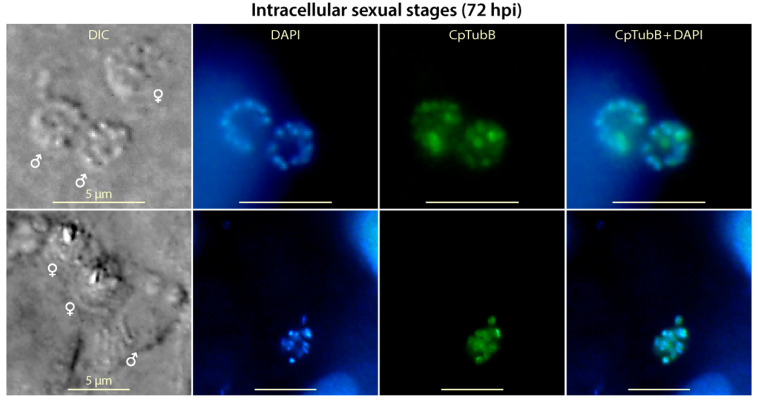
IFA detection of tubulin-based structures in intracellular *Cryptosporidium parvum* in sexual developmental stages at 72 h post-infection (hpi). Both nuclei (blue) and tubulin structures (green) were detectable in the microgametes (marked by male signs) by 4′,6-diamidino-2-phenylindole (DAPI) and anti-CpTubB antibody, but not in the macrogametes (marked by female signs). Note it is characteristic to *C. parvum* that the nuclei of macrogametes were unable to be stained by DAPI. DIC, differential interference contrast microscopy.

Most of the intracellular parasites entered sexual developmental stage at 72 hpi, in which only the nuclei of the microgametes (male gametes), but not those of the large macrogametes (female gametes), could be stained by DAPI (Figure 6). At this stage of development, the anti-CpTubB antibody clearly labeled small dots in microgametes that differed from the long and short filaments in the asexual stages. The morphology of the tubulin-based structures in microgametes could not be well resolved again due to the limitation of epifluorescence microscopy, but it agreed with a previously reported observation that microtubules in microgametes were present as an array of filaments around nuclei by super-resolution structured illumination microscopy (note only tubulin structures in the microgamete stage was presented in this study) [35]. Noticeably, the anti-CpTubB antibody failed to label any structures in the macrogametes, suggesting that tubulin-based structures was absent in the female gametes in *C. parvum*. Similarly, microtubules were also not observed in the macrogametes from other apicomplexans such as *T. gondii* [36].

### 3.7. Lack of Other α- and β-tubulin Orthologs for Alternative Microtubules in the Cryptosporidium Genomes

Because multiple subpellicular microtubules seen in other apicomplexans were undetectable using the affinity-purified anti-CpTubB antibody in this study, we attempted to search for other potential tubulin orthologs from the available *Cryptosporidium* genomes. We found that the three tubulin genes, i.e., α-, β- and γ-tubulin sharing an evolutionarily common ancestor, were all present in single copies in both intestinal and gastric *Cryptosporidium* species, which differs from other apicomplexans that might possess two to three copies of α- and/or β-tubulin genes (Table 2). However, only a single copy of γ-tubulin gene responsible for the nucleation of microtubules was found in all apicomplexans based on the genomic data available today.

Bayesian inference-based phylogenetic analysis showed that α-tubulins were more diverse, forming three clusters: cluster 1 included all species under analysis, cluster 2 contained cystic coccidia and piroplasmids, and cluster 3 contained only cystic coccidia (Figure S1). On the other hand, β-tubulin sequences were much less diverse, and the two isoforms of cystic coccidia (e.g., *T. gondii*) were present within the same cluster, indicating that they evolved from a gene duplication after the split of the cystic coccidia from the intestinal coccidia. The data-mining of the genomes and phylogenetic analysis confirmed the absence of additional tubulin orthologs to form alternative tubulin-based structures in *Cryptosporidium* parasites that might be missed by the labeling of the anti-CpTubB antibody used in this study.

## 4. Conclusions

*Cryptosporidium parvum* possesses only two rigid tubulin-based structures in the sporozoites and merozoites including a long “backbone” microtubule and a short central microtubule, which differs strikingly from other apicomplexans that typically possess an array of subpellicular microtubules in their motile stages, such as *T. gondii*. The microtubules are present in pairs in intracellularly developing meronts, showing as fluorescence-dense structures in microgametes, but undetectable in macrogametes.

## Figures and Tables

**Table 1:** Length of the two microtubules in sporozoites treated with colchicine or paclitaxel during the excystation process.

Treatment Group *	Control	Colchicine (50 μM)		Paclitaxel (50 μM)	
	Mean ± SD (μm)	Mean ± SD (μm)	*p*-Value ^†^	Mean ± SD (μm)	*p*-Value ^†^
Long microtubule	4.13 ± 0.41	4.28 ± 0.36	0.2079	4.33 ± 0.33	0.0683
Short microtubule	1.54 ± 0.38	1.47 ± 0.34	0.6673	1.55 ± 0.33	0.9985

* All treatment groups including the control containing 1% dimethyl sulfoxide (DMSO). ^†^ Adjusted *p*-values derived by Dunnett’s multiple comparison test (vs. control; *n* = 30).

**Table 2:** Number of tubulin subunit isoforms for the major taxonomic groups in the alveolates.

Taxonomic Group	Tubulin Subunit		
	Alpha	Beta	Gamma
Cryptosporidia	1	1	1
Intestinal coccidia	1	1–2	1
Cystic coccidia	3	2	1
Plasmodia	2	1	1
Piroplasmids	2	1	1
Gregarines	2	2	1

## Supplementary Materials

Supplementary materials can be found at https://www.mdpi.com/article/10.3390/microorganisms9091921/s1. Figure S1: Orthologs and phylogenetic analysis of α-, β- and γ-tubulin orthologs in the apicomplexan parasites.
