# Supplementary material for: Unique Tubulin-Based Structures in the Zoonotic Apicomplexan Parasite Cryptosporidium parvum
Source: Microorganisms. 2021 Sep 10;9(9):1921. doi: 10.3390/microorganisms9091921 (PMC8464796; doi:10.3390/microorganisms9091921)
Supplement: Supplementary file 1 [file microorganisms-1355617-supplementary.pdf › microorganisms-1355617-supplementary.pdf]

Figure S1. Orthologs and phylogenetic analysis of α-, β- and γ-tubulin orthologs in the apicomplexan parasites

A

Number of tubulin subunit isoforms

Taxonomic group	Tubulin subunit Alpha	Beta	Gamma
Cryptosporidia	1	1	1
Intestinal coccidia	1	1-2	1
Cystic coccidia	3	2	1
Plasmodia	2	1	1
Piroplasmids	2	1	1
Gregarina	2	2	1

B

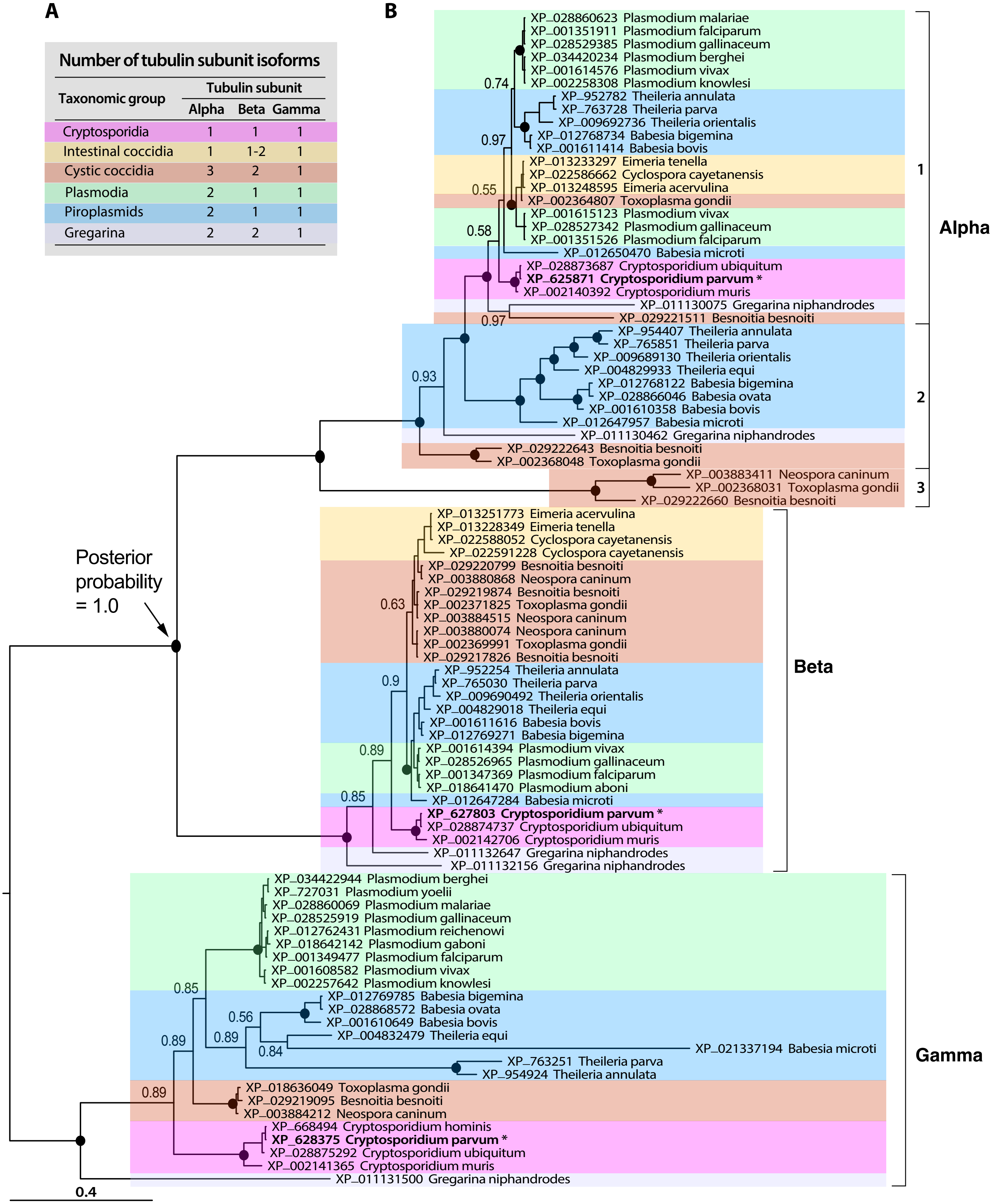

A) Number of isoforms of individual tubulin subunits identified from the genomes of major taxonomic groups of apicomplexans. **B)** Consensus tree inferred from α-, β- and γ-tubulin proteins from representative apicomplexan species by Bayesian inference using a mixed model for amino acid substitution rates with the consideration of invariable sites and four rates of gamma-distribution across the sites (arithmetic mean of log likelihood = −15257.76). Tree was routed using γ-Tubulin sequences as the outgroup. Posterior analysis showed that Jones amino acid substitution model was the most fit model with 100% posterior probability. Numbers at the major nodes indicate proportion support by posterior probability, in which nodes with 100% support were indicated by black round dots. Some species names are colored for better visualization and comparison. Bar indicates amino acid substitution rate.
